# Identifying Risk Factors for Re-admission: A Service Evaluation from an Adult Inpatient Mental Health Unit

**DOI:** 10.1192/bjo.2021.201

**Published:** 2021-06-18

**Authors:** Kirsty Ward, Suveera Prasad

**Affiliations:** Rotherham, Doncaster and South Humber NHS Foundation Trust

## Abstract

**Aims:**

To identify risk factors for re-admission to an acute inpatient general adult mental health ward. There is need to ensure that mental health services adapt to the increasing demand for inpatient beds

**Method:**

We conducted a single centre retrospective analysis of electronic records of 85 discharges from an adult mental health unit from 4th March 2019 – 5th August 2019. We collected information on demographics, admission details, substance use, forensic history, diagnosis as per the International Classification of Diseases 10th Edition (ICD-10), and discharge details and compared two cohorts; those re-admitted within three months of discharge and those who were not. Odds ratio (OR), 95% confidence intervals (CI) and p values were calculated where possible.

**Result:**

Among seventeen service users who were re-admitted within the three month period there were nine women and eight men. There was no difference in ethnicity, employment or marital status. The mean length of admission for those readmitted was 48.2 days (range 1–140 days) and 47.1 days (range 1–350 days) for those who were not readmitted. Certain features were more prevalent among the readmitted group including forensic history (58.8% [10] vs 26.5% [18], OR 3.97, CI 1.31–11.9, p value 0.007), substance misuse history (70.6% [12] vs 55.9% [38], OR 1.89, CI 0.60–5.97, p value 0.138), previous contact with mental health services (100% [17] vs 76.5% [52]) and the rate of detention under the Mental Health Act at point of admission (76.5% [13] vs 66.2% [45], OR 1.66, CI 0.49, 5.67, p value 0.209).

Among those readmitted, a diagnosis of emotionally unstable personality disorder (17.6% [3] vs 10.3% [7], OR 1.87, CI 0.43,-8.14, p values 0.203) and substance misuse disorder (41.2 % [7] vs 17.6 % [12], OR 3.27, CI 1.04–10.31, p value 0.218) were more prevalent. They were more likely to use illicit substances whilst they were an inpatient (23.5% [4] versus 7.6% [5], OR 3.88, CI 0.92–16.43, p value 0.033) and to be involved in police incidents (35.3% [6] versus 17.6% [12], OR 2.55, CI 0.79–8.23, p value 0.059).

**Conclusion:**

Our trends demonstrate that people with substance misuse, emotionally unstable personality disorder and forensic history are more likely to be readmitted to an adult mental health inpatient unit. They were more likely to misuse illicit substances and be involved with police during admission.

